# Influencing factors of hidden blood loss after primary total hip arthroplasty through the posterior approach: a retrospective study

**DOI:** 10.1186/s12891-023-06716-z

**Published:** 2023-07-17

**Authors:** Lijun Cai, Liyile Chen, Chengcheng Zhao, Qiuru Wang, Pengde Kang

**Affiliations:** Department of Orthopedics, Orthopedic Research Institute, West China Hospital, Sichuan University, No. 37 Guoxue Road, Chengdu, 610041 Sichuan People’s Republic of China

**Keywords:** Total hip arthroplasty, Hidden blood loss, Influencing factors, Perioperative management

## Abstract

**Background:**

Total hip arthroplasty (THA) is an excellent treatment for the end-stage hip disease, and perioperative blood management strategies have been effectively applied to this procedure. However, many patients still experience anemia after the operation, which is usually overlooked by orthopedic surgeons due to the hidden blood loss (HBL) in the perioperative period. Therefore, the objective of this study was to evaluate HBL in patients undergoing primary THA using the posterior approach and to explore its influencing factors.

**Methods:**

A retrospective analysis of 707 patients who underwent primary THA through the posterior approach was conducted in our hospital from January 2020 to January 2022. By applying Gross’s and Nadler’s formula, the HBL was calculated. Six quantitative variables (age, body mass index, surgical duration, albumin loss, preoperative hemoglobin, and hemoglobin loss) as well as four qualitative variables (gender, American Society of Anesthesiologists class, major preoperative diagnosis, and hypertension) of patients were analyzed using multivariate linear regression.

**Results:**

The HBL was recorded at 700.39 ± 368.59 mL. As a result of multivariate linear regression analysis, it was determined that body mass index, surgical duration, and hemoglobin loss were all significant risk factors for HBL, whereas preoperative hemoglobin was considered a protective factor. It has been demonstrated that HBL is not significantly correlated with age, albumin loss, gender, ASA class, or major preoperative diagnosis, but it also did not differ from HBL by hypertension.

**Conclusions:**

Hidden blood Loss (HBL) in patients after primary total hip arthroplasty (THA) using the posterior approach is large and significant. When optimizing the perioperative management of THA, orthopedic surgeons should keep in mind HBL and its influencing factors, especially for patients with high body mass indexes, long surgical durations, and low preoperative hemoglobin levels.

**Trial registration:**

This study was registered in the Chinese Clinical Trial Registry (ChiCTR2100053888) in 02/12/2021, http://www.chictr.org.cn.

## Introduction

A total hip arthroplasty (THA), also known as the “operation of the century” [1], is an excellent treatment for the end-stage hip disease that relieves hip pain and restores range of motion, therefore improving the patient’s quality of life and ability to perform. A significant improvement has been made in the length of stay, complications, and readmissions of patients following THA as a result of the development of Enhanced Recovery After Surgery (ERAS) [2, 3]. Blood loss is the most frequent problem in surgery, and massive blood loss is a well-recognized risk factor for a high rate of death, complications, and the use of healthcare resources [4, 5]. Although perioperative blood management strategies have been effectively implemented in THA, and the intraoperative blood loss and postoperative drainage volume are well controlled, many patients continue to develop anemia after the operation to varying degrees, and in some patients severe anemia occurs, which appears to be inconsistent with the visible blood loss [5]. Most surgeons overlook the invisible bleeding, such as bleeding volume occurring in tissue spaces and body cavities [6–8]. Therefore, this separated blood volume should also be included in the perioperative blood management strategies.

The concept of hidden blood loss (HBL) was proposed by Sehat KR et al. in 2000 [9], covering blood loss excluding visible blood loss (VBL) such as intraoperative blood loss (IBL) and postoperative drainage volume. In recent years, HBL had received increasing attention with advances in research on perioperative blood loss. Researchers had found that HBL in the perioperative period of various surgical procedures accounted for a greater proportion than thought in terms of total blood loss [6, 8, 10, 11]. However, to our knowledge, few studies have reported the influencing factors of HBL after primary THA through the posterior approach.

The present retrospective study was conducted to evaluate HBL in patients undergoing primary THA through the posterior approach and to explore its influencing factors. This study hypothesized that by optimizing these factors, it will be useful to reduce perioperative bleeding, accelerate the overall rehabilitation progress, and reduce the occurrence of long-term adverse consequences.

## Materials and methods

### Patients

This retrospective study enrolled patients who underwent primary unilateral THA through the posterior approach at West China Hospital, Sichuan University, from January 2020 to January 2022. The inclusion criteria were patients with complete demographic and clinical data, no earlier hip surgery on the affected side, and an American Society of Anesthesiologists (ASA) score of I-III. Patients with bilateral THA, incomplete data, blood, or albumin transfusions during hospitalization, and hematologic diseases were excluded.

### Perioperative Management

All primary THA procedures were performed, with the patient under general anesthesia, by five senior joint surgeons at the institution with the posterior approach from January 2020 to January 2022. The patient was placed in a lateral decubitus position on a standard operating table after anesthesia. A 10–15 cm curved incision was utilized on the posterior-lateral side of the hip through the apex of the greater trochanter. The skin and subcutaneous tissues were incised, the gluteus maximus and deep fascia were bluntly separated, the external rotators was cut, and the hip capsule was exposed and incised. After flexion, internal retraction and internal rotation to dislocate the hip joint, the rest of the operation was performed as in the literature [12–14]. In all surgeries, the cementless total hip prosthesis (Depuy Synthes, USA) including acetabular and femoral components, was used. After primary THA, a drainage tube was not routinely inserted unless the surgical wound was bleeding a lot.

The patients received tranexamic acid (20 mg/kg) intravenously 5–10 min before the skin incision and 1 g of tranexamic acid 3 and 6 h after surgery. When patients return to the ward after surgery a cold pack was applied around the surgical incision for 12 h. As an infection prevention measure, 2 g of cefazolin sodium were administered intravenously during the surgery and continued for 24 h postoperatively. At this center, a standard protocol was administered having physical and drug prophylaxis to prevent venous thromboembolism (VTE). Postoperatively, the same group of orthopedic surgeons to begin passive and active rehabilitation exercises, ankle pump exercise and foot pump system application within 24 h after surgery, lower limb strength training starting after fully awakening from anesthesia, and walking with full or part weight bearing as soon as possible after X-ray examination for review that often happened on the first postoperative day. Half dose (0.2 mL, or 2000 IU aXa; Clexane, France) of low molecular weight heparin (LMWH) was administered subcutaneously 8 h after surgery, followed by a full dose (0.4 mL) every 24 h until discharge from the hospital, which was immediately discontinued if severe wound bleeding or subcutaneous bleeding and other conditions were not suitable for anticoagulation. In addition, all patients continued to receive oral administration of rivaroxaban (10 mg, once a day) for 2 weeks after discharge to prevent VTE. For analgesia, intraoperative periarticular infiltration analgesia and postoperative oral non-steroidal anti-inflammatory drugs (NSAIDs) such as celecoxib (200 mg, twice daily) were the routine procedures. If the pain was severe, 5 mg of morphine hydrochloride was prescribed subcutaneously to remedy the analgesia.

### Outcome measures

The electronic medical record system could be used to collect general demographic data on all patients including gender, age, height, weight, body mass index (BMI) and various data during hospitalization such as major preoperative diagnosis, comorbidities, American Society of Anesthesiologists (ASA) classification, the duration of surgery, estimated intraoperative blood loss (IBL), postoperative drainage volume, postoperative length of hospital stay (LOS) along with complete laboratory data consisted of patient’s levels of hematocrit (Hct), hemoglobin (Hb), and plasma albumin (Alb) levels within 72 h before surgery and 72 h postoperatively.

The primary outcome measure was hidden blood loss (HBL), which can be defined as total blood loss (TBL) minus visible blood loss (VBL) [15, 16]. The secondary outcome measures comprised TBL, VBL, IBL, and reduction in Hb and Alb. VBL was the sum of IBL and postoperative drainage volume. The IBL was equal to the volume of liquid in the negative pressure suction device minus the volume of flushing saline, plus the net weight gain of the gauze, which can be collected from the anaesthetic records. The reduction in Hb and Alb levels was obtained by subtracting the lowest value within 3 days after surgery from the preoperative level. Other outcome measures will be described in detail subsequently.

The TBL was calculated from the Gross formula [17]: TBL (mL) = the patient’s blood volume (PBV) × (preoperative Hct - postoperative Hct) / average of Hct, where the average of Hct is the mean of preoperative Hct and postoperative Hct. Likewise, calculation of PBV was also required the Nadler’s formula [18]: PBV (mL) = [k1 × height (m)^3^ + k2 × weight (kg) + k3] × 1000, where k1 = 0.3669, k2 = 0.03219, and k3 = 0.6041 for male patients, and k1 = 0.3561, k2 = 0.03308, and k3 = 0.1833 for female patients.

### Statistical analysis

SPSS v23.0 software (IBM, USA) was applied for all statistical analyzes. The quantitative data were expressed as means and standard deviations (SDs), while the qualitative data were expressed as frequencies and percentages. Multivariate linear regression analysis was used to identified the influencing factors related to HBL, including six quantitative variables (age, BMI, surgical duration, Alb loss, preoperative Hb and Hb loss) and four qualitative variables (gender, ASA class, major preoperative diagnosis and hypertension). Among the qualitative variables, male, ASA class 1, rheumatoid arthritis, and non-hypertension was set as “0”. A positive coefficient indicates a positive effect of the factor on the dependent variable (HBL), while the opposite is a negative effect. A P-value < 0.05 was considered a statistically significant difference.

## Results

Between January 2020 and January 2022, 731 patients underwent primary THA with the posterior approach at this hospital, of whom 24 did not satisfy the inclusion criteria and were therefore excluded. Ultimately, 707 of the eligible cases were included in the analysis. The mean age was 54.7 ± 13.3 years and the average BMI was 23.93 ± 3.22 kg/m^2^, which contained 319 males and 388 females. Drainage tubes were placed in 17 patients with an average drainage volume of 171mL. The specific baseline characteristics are represented in Table 1.

**Table 1:** Patients’ demographic information

Baseline characteristic	All patients (n = 707)
Demographic characteristics	
Age (years)	54.70 ± 13.30
Gender, M/F	319/388
Height (cm)	160.57 ± 8.61
Weight (kg)	61.78 ± 10.16
BMI (kg/m2)	23.93 ± 3.22
Diagnosis, N (%)	
ONFH	298 (42.15%)
DDH	251 (35.50%)
OA	114 (16.12%)
AS	25 (3.54%)
RA	19 (2.69%)
Comorbidities, N (%)	
Hypertension	177 (25.04%)
Type 2 diabetes	41 (5.80%)
CHD	96 (13.58%)
COPD	7 (0.99%)
Chronic bronchitis	24 (3.39%)
CKD	14 (1.98%)
Chronic gastritis	13 (1.84%)
Anemia	39 (5.52%)
SLE	11 (1.56%)
Gout	16 (2.26%)
Osteoporosis	230 (32.53%)
ASA class, N (%)	
I	10 (1.42%)
II	590 (83.45%)
III	107 (15.13%)
Surgical duration (minutes)	65.51 ± 19.25
Postoperative LOS (day)	2.52 ± 0.85

BMI, body mass index; ONFH, osteonecrosis of femoral head; DDH, developmental dysplasia of the hip; OA, osteoarthritis; AS, ankylosing spondylitis; RA, rheumatoid arthritis; CHD, coronary heart disease; COPD, chronic obstructive pulmonary disease; CKD, chronic kidney disease; SLE, systemic lupus erythematosus; ASA, American Society of Anesthesiologists; LOS, length of stay

The data for preoperative Alb, preoperative Hb, preoperative Hct, Alb loss, Hb loss, IBL, VBL, HBL, and TBL are shown in Table 2. The mean Alb loss was 8.80 ± 3.37 g/L, the mean Hb loss was 25.02 ± 11.10 g/L, the mean IBL was 109.70 ± 42.35 mL, the mean HBL was 700.39 ± 368.59 mL and the mean TBL was 814.19 ± 370.41 mL.

**Table 2:** Clinical Results in the Patients

Parameters	Values
Preoperative Alb (g/L)	45.89 ± 3.18
Preoperative Hb (g/L)	137.19 ± 1 6.20
Preoperative Hct (L/L)	0.42 ± 0.04
Alb loss (g/L)	8.80 ± 3.37
Hb loss (g/L)	25.02 ± 11.10
Intraoperative blood loss (mL)	109.70 ± 42.35
Visible blood loss (mL)	113.81 ± 56.55
Hidden blood loss (mL)	700.39 ± 368.59
Total blood loss (mL)	814.19 ± 370.41

Alb, albumin; Hb, hemoglobin; Hct, hematocrit

Multiple linear regression analysis was used to identify the relationship between the influencing factors and HBL, which included six quantitative variables and four qualitative variables, as previously mentioned. According to Table 3, it was found that BMI (p < 0.001), surgical duration (p < 0.001) and Hb loss (p < 0.001) had a positive effect on HBL. Compared to patients with standard weight, the higher the BMI, the more the perioperative HBL. In addition, perioperative HBL increased with prolongation of the surgical duration, as the patients with an increase in Hb loss. On the contrary, the amount of preoperative Hb (p < 0.001) was negatively related to HBL. Patients with a lower preoperative Hb had significantly higher perioperative HBL. Age (p = 0.694), Alb loss (p = 0.665), gender (p = 0.581), ASA class (p = 0.758), major preoperative diagnosis (p = 0.449) and hypertension (p = 0.562) did not significantly correlate with HBL.

**Table 3:** Multivariate linear regression for factors related to HBL

	Unstandardized B	standardized B	t	P	VIF
Quantitative variables					
Age (year)	-0.156	-0.006	-0.394	0.694	1.173
BMI (kg/m2)	11.594	0.101	5.906	< 0.001	1.127
Surgical duration (minutes)	1.046	0.055	3.307	< 0.001	1.045
ALB loss (g/L)	1.015	0.009	0.433	0.665	2.647
Preoperative Hb (g/L)	-1.675	-0.074	-4.062	< 0.001	1.260
Hb loss (g/L)	31.550	0.950	42.217	< 0.001	1.941
Qualitative variables					
Gender (female)	-151.973	-0.205	-0.557	0.581	1.000
ASA class (1)					
2	-35.985	-0.036	-0.308	0.758	9.929
3	-168.644	-0.164	-1.394	0.164	9.929
Preoperative diagnosis (RA)					
ONFH	65.979	0.088	0.757	0.449	9.652
DDH	3.770	0.005	0.043	0.966	9.165
OA	3.140	0.003	0.034	0.973	5.871
AS	13.329	0.007	0.119	0.905	2.234
Hypertension	18.559	0.022	0.580	0.562	1.000

HBL, Hidden blood loss

## Discussion

In this retrospective study, we included multiple potentially influencing factors for HBL and found by multiple linear regression analysis that BMI, surgical duration, and Hb loss were positively associated with HBL, while preoperative Hb was negatively associated with HBL and the association was statistically significant, whereas age, Alb loss, gender, ASA class, major preoperative diagnosis, and hypertension did not seem to be an independent risk factor for HBL. Perioperative bleeding is still a predominant risk factor in surgery, associated with high mortality, complications, and unsatisfactory outcomes [4]. Surgeons usually concentrate on the VBL including IBL and postoperative drainage and regard them as the total perioperative blood loss since their visualization and direct measurement, while HBL is often overlooked and makes the TBL be underestimated [19, 20]. It was not until 2000 that Sehat et al. described this category of undetectable blood loss called HBL [9], which had gradually aroused extensive attention from surgeons. For the source of HBL, there are various discussions and no firm conclusion has yet been reached. Sehat et al. [9] proposed that HBL originated from extravasation of blood into the tissues, residual blood in the surgical cavity or dead space, and hemolysis caused by reinfusion with unwashed filtered blood. Ogura et al. [6] suggested that, besides the aforementioned sources, inaccurate measurement of IBL and poorly functioning drains were also possible sources of HBL. In addition, Bao et al. [21] considered that HBL is because of erythrocyte injury and hemoglobin peroxidation, in which free fatty acids (FFA) play an important role by stimulating neutrophil production of reactive oxygen species.

The volume of HBL is not negligible during the whole perioperative period and often exceeds the expectations of doctors. Regarding patients undergoing total knee arthroplasty, SHEN et al. [22] retrospectively analyzed 108 patients and detected HBL as (51.5 ± 9.3) % of TBL, and Sehat et al. [9]prospectively studied 63 patients and observed that HBL accounted for 50% of TBL. In this study, by retrospective analysis of 707 patients who underwent primary THA using the posterior approach, the amount of Hb loss and Alb loss was 25.02 ± 11.10 g/L and 8.80 ± 3.37 g/L, respectively. Meanwhile, the amount of HBL was approximately 700.39 ± 368.59 mL, and the TBL was approximately 814.19 ± 370.41 mL. In the arthroplasty center, only 17 of the 707 patients had drainage tubes placed, with each draining a small volume, averaging only 171 mL, contributing to the IBL approaching the volume of the VBL. According to earlier diagnostic criteria for anemia [23], 221 men and 343 women among these patients developed varying degrees of postoperative anemia.

However, it is unclear which risk factors may influence HBL since the specific pathogenesis of HBL still is controversial. WANG et al. found from a retrospective analysis of patients after intramedullary nail fixation of extra-articular tibial fractures that gender, surgical duration, and diameter of the medullary cavity independently influenced HBL [24]. By retrospectively analyzing patients who underwent posterior lumbar fusion, Wen et al. revealed that multilevel fusion, surgical time, and fibrinogen level were independent risk factors of HBL, while age and postoperative complications were not significantly associated with HBL [25]. This study suggested that the higher the patient’s BMI, the more HBL the patient has in the perioperative period. Lei et al. retrospectively analyzed the factors that influence HBL in patients undergoing posterior lumbar fusion and found that when the patient’s BMI > 24 kg/m^2^ significantly increased perioperative HBL. They hypothesized that obese patients under anesthesia would alter cardiorespiratory physiology such as lower pulmonary compliance, and overcoming these alterations would increase ventilation pressures, which would increase venous pressures and increase venous bleeding [26]. Bowditch et al. concluded that obese patients with a large area of subcutaneous fat exposure have a higher blood loss [27]. Similarly, Ogura et al. believed that patients with a higher BMI may have more space for extravasation of blood [6]. Furthermore, as in most research on the factors influencing HBL, this research revealed that surgical duration is strongly correlated with HBL of patients, and the HBL increases significantly with the increase in surgery duration. The increased amount of blood exuding from the surgical area when the operation takes longer, which inevitably leads to a higher amount of bleeding, including HBL. In addition, substantial release and removal of soft tissues increases the risk of postoperative bleeding and more space for extravasation of blood. Moreover, the fact that intraoperative blood may coagulate in the third interval and cannot be drained, and is often overlooked [11, 24, 26, 28]. An earlier study showed that preoperative Hb was a risk factor for HBL in patients with proximal femur fractures, where patients with lower preoperative Hb had higher HBL [29]. The authors suggested that these patients with preexisting preoperative anemia may be in a frail state, so that the entire body’s hemoglobin reserve and ability to withstand and recover from major surgical insult are decreased. Additionally, it has been suggested that lower preoperative hemoglobin predisposes to a higher rate of transfusion, which is a well-established risk factor for HBL, with hemolysis as a possible cause [24, 30, 31]. Similarly, this study found that preoperative Hb and losing Hb during perioperative periods were risk factors for HBL in patients undergoing primary THA through the posterior approach.

Earlier studies demonstrated that hypoalbuminemia was also considered associated with HBL [32]. It is well known that decreased plasma albumin leads to reduced plasma colloid osmotic pressure, resulting in plasma extravasation, which would increase HBL, and some scholars [21] also proposed that albumin could decrease HBL by combining with FFA, attenuating the toxicity of FFA and reducing oxidative damage to erythrocytes. However, patients with hypoalbuminemia were eliminated when screening surgical patients in our medical center, so this study did not include hypoalbuminemia as a possible influencing factor for HBL.

There were several limitations in this study. First, this study had a few patients and was a single-center study with insufficient evidence, which requires more prospective studies with a larger data volume from multicenter to optimize or modify the conclusions for better promotion in the clinic. Furthermore, the specific time point for completion of body fluid transfer in postoperative patients is still inconclusive for now and Sehat et al. [33] suggested that hemodynamic stability was basically completed on the second to third day after operation, thus the use of the laboratory results on the second or third day after operation to calculate bleeding volume in this study may not be the optimum time for measurement. Finally, patients who received blood or albumin transfusions during hospitalization were not included in this study and their effect on HBL was not assessed.

## Conclusions

The HBL in patients after primary THA through the posterior approach is sizeable and significant, which is much higher than expected by orthopedic surgeons and usually causes patients to develop postoperative anemia that is inconsistent with the VBL. More importantly, BMI, surgical duration, preoperative Hb and Hb loss are the influencing factors of HBL in patients after primary THA through the posterior approach, where BMI, surgical duration, and Hb loss are risk factors, while preoperative Hb is a protective factor. Orthopedic surgeons should focus on HBL and its influencing factors when optimizing the perioperative management of THA, especially for patients with high BMI, long surgical duration and low preoperative Hb.

## Data Availability

The datasets used and/or analysed during the current study are available from the corresponding author on reasonable request.

## Abbreviations

THA: Total hip arthroplasty
HBL: Hidden blood loss
ERAS: Enhanced recovery after surgery
TBL: Total blood loss
VBL: Visible blood loss
IBL: Intraoperative blood loss
ASA: American society of anesthesiologists
VTE: Venous thromboembolism
LMWH: Low molecular weight heparin
NSAIDs: Non-steroidal anti-inflammatory drugs
BMI: Body mass index
LOS: Length of hospital stay
Hct: Hematocrit
Hb: Hemoglobin
Alb: Albumin
PBV: Patient’s blood volume
SDs: Standard deviations
FFA: Free fatty acids
